# Tracking the evolution of an extensively drug-resistant cross-border *Mycobacterium tuberculosis* cluster, Europe, January 2016 up to August 2025: implications for European surveillance

**DOI:** 10.2807/1560-7917.ES.2025.30.46.2500838

**Published:** 2025-11-20

**Authors:** Francesca Saluzzo, Chiara Sepulcri, Alma Zinola, Federico Di Marco, Luca Ragazzoni, Marco Rossi, Luigi Codecasa, Daniela Maria Cirillo

**Affiliations:** 1 CRIMEDIM - Center for Research and Training in Disaster Medicine, Humanitarian Aid and Global Health, Università del Piemonte Orientale Novara, Italy; 2 Department of Immunology, Transplantation and Infectious Diseases, IRCCS San Raffaele Scientific Institute, Milan, Italy; 3 Division of Infectious Diseases, Department of Health Sciences, University of Genova, Genova, Italy; 4 Regional TB Reference Clinic, Villa Marelli Institute, Niguarda Hospital, Milan, Italy

**Keywords:** Drug-resistant Tuberculosis, Pretomanid resistance, Cross-border transmission, XDR-TB transmission

## Abstract

The emergence and spread of an extensively drug-resistant (XDR) *Mycobacterium tuberculosis* lineage 4.8 cluster in Europe raises public health concerns. First reported in 2020 across Romania, Italy and the United Kingdom, this cluster progressed from multidrug-resistant (MDR) and pre-extensively drug-resistant (pre-XDR) to XDR, including resistance to pretomanid. Evidence of ongoing local transmission is available for Italy, where 10 cases were reported from 2021 to 2025. Strengthened whole genome sequencing-based surveillance is needed to inform timely, coordinated public health responses.

In summer 2025, three tuberculosis cases belonging to a known European multidrug-resistant tuberculosis (MDR-TB) cluster were identified in Italy. They showed an extensively drug-resistant (XDR) profile, and one also had additional resistance to pretomanid. To investigate cluster transmission, extent and evolution, we analysed all MDR/pre-XDR and XDR sequences submitted to the European Centre for Disease Prevention and Control (ECDC) from 2016 to 2024 and integrated these with data from the Italian MDR-TB surveillance system up to August 2025.

## Genomic surveillance of drug-resistant tuberculosis

The surveillance and management of drug-resistant tuberculosis (TB) pose an ongoing challenge to elimination efforts in the European Union/European Economic Area (EU/EEA) [1,2]. Previous genomic surveillance in the context of the EUSeqMyTB project [3], supported by the ECDC, identified a drug-resistant *Mycobacterium tuberculosis* (MTB) lineage 4.8 cross-border cluster, named snpCL1, with cases detected in Romania, Italy and the United Kingdom (UK). The cluster, identified through whole genome sequencing (WGS), accounted for 34 cases isolated between 2016 and 2020. Seventeen were multidrug-resistant (MDR) cases, 10 pre-extensively drug-resistant (pre-XDR) cases and 7 XDR cases [4], classified according to the most recent World Health Organization (WHO) definition [5]. Since 2021, 10 additional XDR cases have been identified through WGS, increasing the total to 44. The most recent three cases were all detected in the same northern Italian city between June and August 2025. These findings show that the snpCL1 XDR clone continues to circulate and has developed into an XDR pattern, with two cases linked to resistance to pretomanid, raising urgent public health concerns.

## Tracing snpCL1: cluster identification and transmission patterns

We combined European data and sequences from the ECDC’s European Surveillance System (TESSy) [6] with Italian MDR surveillance data. The final dataset included 2,834 sequences spanning from January 2016 to June 2024 (the latest available data at the time of the request), provided by Austria, Belgium, Bulgaria, Croatia, Czechia, Denmark, Estonia, Finland, France, Germany, Hungary, Ireland, Italy, Lithuania, Latvia, the Netherlands, Norway, Poland, Portugal, Romania, Slovakia, Slovenia, Spain, Sweden and the UK (available up to 2019), plus an additional 50 sequences from the Italian MDR surveillance data from July 2024 to August 2025. Of these, 2,842 sequences (98.5%) met quality criteria (breadth coverage 8x > 98% and coverage depth > 30) for subsequent analysis.

**Figure 1:**
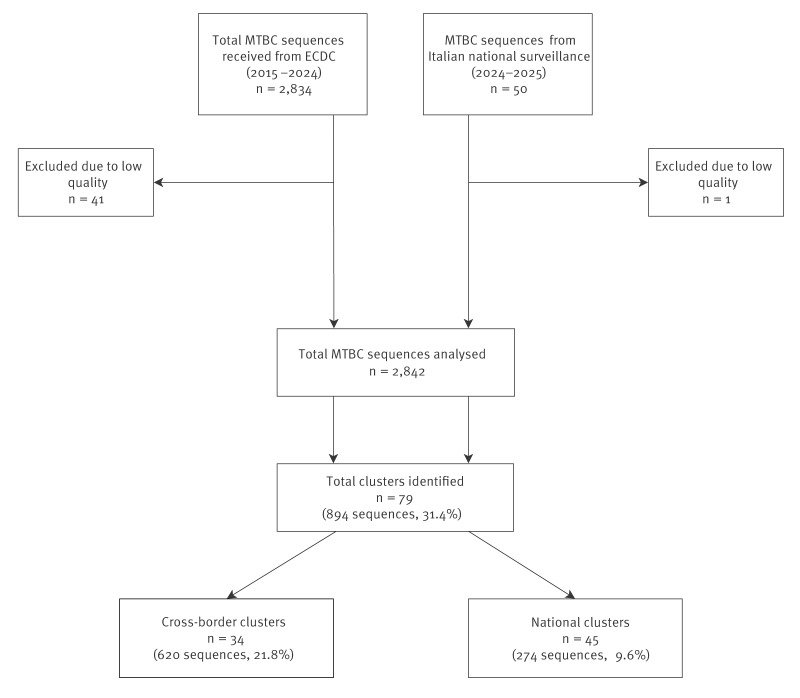
Flow diagram of *Mycobacterium tuberculosis* sequences included in the analysis and resulting cluster classification, 2016–2025 (n = 2,842)

ECDC: European Centre for Disease Prevention and Control; MTBC: *Mycobacterium tuberculosis* complex.

To reduce computational demands, the relatedness analysis was initially performed using core genome multilocus sequence typing (cgMLST) in Ridom SeqSphere + v10.00 by applying a threshold of ≤ 5 allelic variants to define a transmission cluster. We identified 79 clusters (35 cross-border and 44 national) comprising 894 sequences (Figure 1). A summary of the number of samples per cluster and countries involved is provided in Supplementary Tables S1 and S2. The three XDR strains were part of a cluster of 94 cases diagnosed across four countries: Italy, Romania, the UK and Austria (see Supplementary Figure S1 for details about the number of isolates per country).

Data provided by ECDC based on data reported by EU/EEA Member States on the European Surveillance System (TESSy). One sequence per individual was included in the dataset.

**Figure 2:**
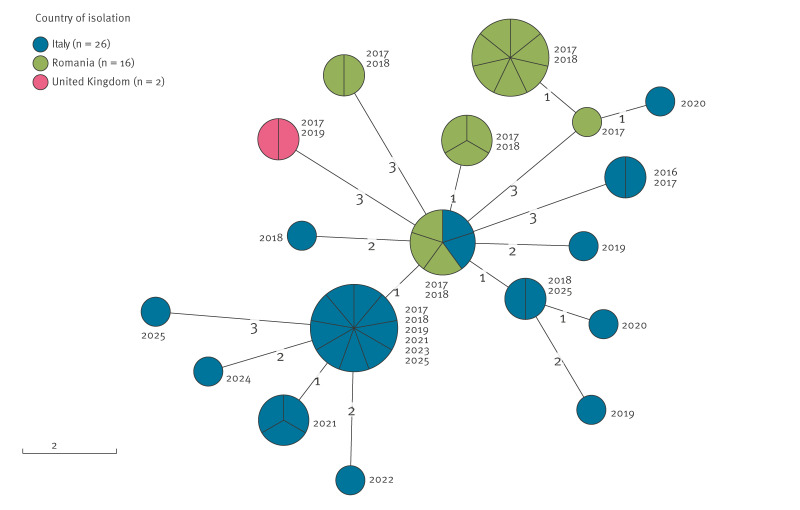
*Mycobacterium tuberculosis* snpCL1 cluster transmission tree, Europe, 2016–2025^a^ (n = 44)

^a^ Data from Romania and United Kingdom are available only from 2016 to 2020.

Given the higher resolution of the single nucleotide polymorphism (SNP)-based analysis than the cgMLST, this cluster was reanalysed using the MTBseq v1.0.3 pipeline and GrapeTree v2.2 [7] to generate and visualise the transmission tree. A SNP threshold of ≤ 5 was employed to assess recent transmission [8]. As a result, the final snpCL1 cluster included 44 isolates collected between 2017 and 2025 (Figure 2).

The analysis was performed with MTBseq. The results were processed using GrapeTree (v2.2) to generate and visualise the final transmission tree. Each circle represents a node within the cluster, marked with the corresponding years of reporting, and is proportionally divided to reflect the number of samples included in that node. The numbers on the connecting lines between circles represent the distance in allelic variants between nodes.

**Figure 3:**
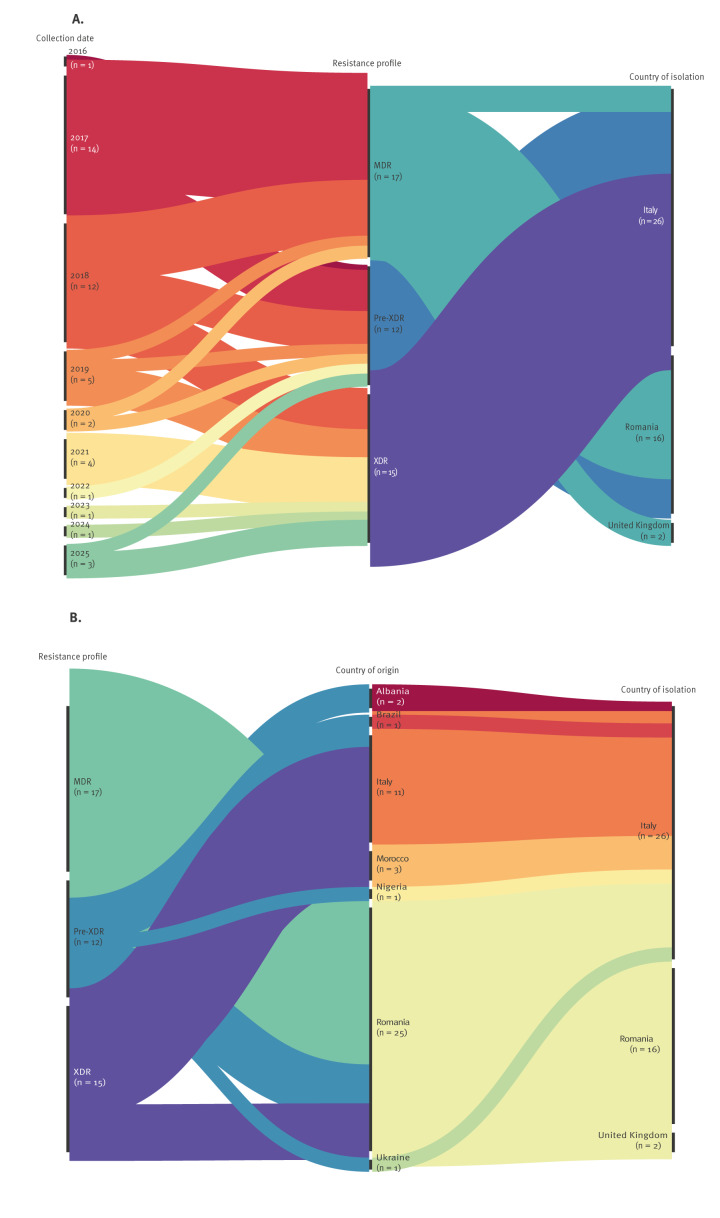
Temporal, geographic distribution and progression of drug resistance profiles among *Mycobacterium tuberculosis* isolates belonging to the snpCL1 cluster, Europe, 2016–2025 (n = 44)

MDR: multidrug-resistant tuberculosis; XDR: extensively drug-resistant tuberculosis.

Most isolates were collected in Italy (n = 26) followed by Romania (n = 16) and the UK (n = 2). The country of origin was known for all 44 cases, with 17 individuals being foreign-born. The majority of isolates were obtained from people originating from Romania (n = 25) and Italy (n = 11), while others came from Morocco (n = 3; 6.8%), Albania (n = 2; 4.5%), Nigeria, Ukraine or Brazil (n = 1 each). Between 2016 and 2019, 34 isolates were identified across Italy, Romania and the UK, suggesting early cross-border transmission followed by local spread (Figure 3, panel A). From 2020 onward, the lack of genomic data from Romania and the UK in TESSy limited the assessment of the cluster’s evolution. The 10 most recent isolates (2021–2025) were detected in Italy among individuals of diverse countries of origin (Figure 3, panel B). Resistance profiles showed a progressive shift from MDR and pre-XDR to exclusively pre-XDR and XDR, indicating increasing resistance within the cluster.

Resistance profiles were determined according to the World Health Organization Catalogue of mutations, version 2 [10].

## Resistance determinants analysis

The samples were analysed using a pipeline developed in-house that integrates MTBseq (v1.0.4) [8] and polishes analysis for structural variations, such as insertions and deletions, with Delly (v1.1.6) [9]. 

Resistance mutations were classified according to the WHO catalogue of mutations, version 2 [10]. The genotypic resistance determinants identified in the snpCL1 cluster are summarised in the Table. All isolates carried the mutations *rpoB* p.Ser450Leu and *katG* p.Ser315Thr, together with the *inhA* promoter variant c.-777C > T, *embB* p.Met306Ile, and *pncA* p.Ala146Val, conferring resistance to all first-line drugs, i.e. rifampicin, isoniazid, ethambutol and pyrazinamide.

**Table:** Analysis of the resistance determinants of snpCL1 cluster according to the World Health Organization mutation catalogue, version 2 [10], Europe, 2016–2025 (n = 44)

Sample ID	Resistance profile	Resistance determinants identified per drug							
		Rifampicin	Isoniazid	Ethambutol	Pyrazinamide	Fluoroquinolones	Bedaquiline	Delamanid	Amikacin
EUST21507	MDR	rpoB_p.Ser450Leu	inhA_c.-777C > T, katG_p.Ser315Thr	embB_p.Met306Ile	pncA_p.Ala146Val	NI	NI	NI	NI
EUST09690	MDR	rpoB_p.Ser450Leu	inhA_c.-777C > T, katG_p.Ser315Thr	embB_p.Met306Ile	pncA_p.Ala146Val	NI	NI	NI	NI
EUST10976	MDR	rpoB_p.Ser450Leu	inhA_c.-777C > T, katG_p.Ser315Thr	embB_p.Met306Ile	pncA_p.Ala146Val	NI	NI	NI	NI
EUST12006	MDR	rpoB_p.Ser450Leu	inhA_c.-777C > T, katG_p.Ser315Thr	embB_p.Met306Ile	pncA_p.Ala146Val	NI	NI	NI	NI
EUST16763	Pre-XDR	rpoB_p.Ser450Leu	inhA_c.-777C > T, katG_p.Ser315Thr	embB_p.Met306Ile	pncA_p.Ala146Val	gyrB_p.Ala504Val	NI	NI	NI
EUST17397	MDR	rpoB_p.Ser450Leu	inhA_c.-777C > T, katG_p.Ser315Thr	embB_p.Met306Ile	pncA_p.Ala146Val	NI	NI	NI	NI
EUST00659	MDR	rpoB_p.Ser450Leu	inhA_c.-777C > T, katG_p.Ser315Thr	embB_p.Met306Ile	pncA_p.Ala146Val	NI	NI	NI	NI
EUST19390	Pre-XDR	rpoB_p.Ser450Leu	inhA_c.-777C > T, katG_p.Ser315Thr	embB_p.Met306Ile	pncA_p.Ala146Val	gyrB_p.Ala504Val	NI	NI	NI
EUST16342	MDR	rpoB_p.Ser450Leu	inhA_c.-777C > T, katG_p.Ser315Thr	embB_p.Met306Ile	pncA_p.Ala146Val	NI	NI	NI	NI
EUST11224	MDR	rpoB_p.Ser450Leu	inhA_c.-777C > T, katG_p.Ser315Thr	embB_p.Met306Ile	pncA_p.Ala146Val	NI	NI	NI	NI
EUST13956	Pre-XDR	rpoB_p.Ser450Leu	inhA_c.-777C > T, katG_p.Ser315Thr	embB_p.Met306Ile	pncA_p.Ala146Val	gyrA_p.Asp94Gly	NI	NI	rrs_n.1401A > G
EUST16797	MDR	rpoB_p.Ser450Leu	inhA_c.-777C > T, katG_p.Ser315Thr	embB_p.Met306Ile	pncA_p.Ala146Val	NI	NI	NI	NI
EUST17292	MDR	rpoB_p.Ser450Leu	inhA_c.-777C > T, katG_p.Ser315Thr	embB_p.Met306Ile	pncA_p.Ala146Val	NI	NI	NI	NI
EUST00154	Pre-XDR	rpoB_p.Ser450Leu	inhA_c.-777C > T, katG_p.Ser315Thr	embB_p.Met306Ile	pncA_p.Ala146Val	gyrA_p.Asp94Tyr	NI	NI	NI
EUST00253	XDR	rpoB_p.Ser450Leu	inhA_c.-777C > T, katG_p.Ser315Thr	embB_p.Met306Ile	pncA_p.Ala146Val	gyrA_p.Asp94Tyr	Rv0678_lof(778990:Del-long);Rv0678_p.Gly25Cys^a^	NI	NI
EUST20429	MDR	rpoB_p.Ser450Leu	inhA_c.-777C > T, katG_p.Ser315Thr	embB_p.Met306Ile	pncA_p.Ala146Val	NI	NI	NI	NI
EUST00140	Pre-XDR	rpoB_p.Ser450Leu	inhA_c.-777C > T, katG_p.Ser315Thr	embB_p.Met306Ile	pncA_p.Ala146Val	gyrA_p.Asp94Tyr	NI	NI	NI
EUST13912	Pre-XDR	rpoB_p.Ser450Leu	inhA_c.-777C > T, katG_p.Ser315Thr	embB_p.Met306Ile	pncA_p.Ala146Val	gyrB_p.Ala504Val	NI	NI	NI
EUST00190	Pre-XDR	rpoB_p.Ser450Leu	inhA_c.-777C > T, katG_p.Ser315Thr	embB_p.Met306Ile	pncA_p.Ala146Val	gyrA_p.Asp94Tyr	Rv0678_p.Gly25Cys^a^	NI	NI
EUST14460	MDR	rpoB_p.Ser450Leu	inhA_c.-777C > T, katG_p.Ser315Thr	embB_p.Met306Ile	pncA_p.Ala146Val	NI	NI	NI	NI
EUST13944	MDR	rpoB_p.Ser450Leu	inhA_c.-777C > T, katG_p.Ser315Thr	embB_p.Met306Ile	pncA_p.Ala146Val	NI	NI	NI	NI
EUST14149	MDR	rpoB_p.Ser450Leu	inhA_c.-777C > T, katG_p.Ser315Thr	embB_p.Met306Ile	pncA_p.Ala146Val	NI	NI	NI	NI
EUST16997	MDR	rpoB_p.Ser450Leu	inhA_c.-777C > T, katG_p.Ser315Thr	embB_p.Met306Ile	pncA_p.Ala146Val	NI	NI	NI	NI
EUST00276	XDR	rpoB_p.Ser450Leu	inhA_c.-777C > T, katG_p.Ser315Thr	embB_p.Met306Ile	pncA_p.Ala146Val	gyrA_p.Asp94Tyr	Rv0678_lof(778990:Del-long)	fbiC_His722Arg^b,c^	NI
EUST09570	XDR	rpoB_p.Ser450Leu	inhA_c.-777C > T, katG_p.Ser315Thr	embB_p.Met306Ile	pncA_p.Ala146Val	gyrA_p.Asp94Tyr	Rv0678_lof(778990:Del-long)	NI	NI
EUST15897	XDR	rpoB_p.Ser450Leu	inhA_c.-777C > T, katG_p.Ser315Thr	embB_p.Met306Ile	pncA_p.Ala146Val	gyrA_p.Asp94Tyr	Rv0678_lof(778990:Del-long)	NI	NI
EUST19363	XDR	rpoB_p.Ser450Leu	inhA_c.-777C > T, katG_p.Ser315Thr	embB_p.Met306Ile	pncA_p.Ala146Val	gyrA_p.Asp94Tyr	Rv0678_lof(779012:Del-1); Rv0678_p.Ala59Val^a^	NI	NI
EUST04520	MDR	rpoB_p.Ser450Leu	inhA_c.-777C > T, katG_p.Ser315Thr	embB_p.Met306Ile	pncA_p.Ala146Val	NI	NI	NI	NI
EUST09473	XDR	rpoB_p.Ser450Leu	inhA_c.-777C > T, katG_p.Ser315Thr	embB_p.Met306Ile	pncA_p.Ala146Val	gyrA_p.Asp94Tyr	Rv0678_lof(778990:Del-long)	NI	NI
EUST24967	MDR	rpoB_p.Ser450Leu	inhA_c.-777C > T, katG_p.Ser315Thr	embB_p.Met306Ile	pncA_p.Ala146Val	NI	NI	NI	NI
EUST01776	Pre-XDR	rpoB_p.Ser450Leu	inhA_c.-777C > T, katG_p.Ser315Thr	embB_p.Met306Ile	pncA_p.Ala146Val	gyrA_p.Asp94Tyr	Rv0678_Arg34Leu^b^	NI	NI
EUST22603	Pre-XDR	rpoB_p.Ser450Leu	inhA_c.-777C > T, katG_p.Ser315Thr	embB_p.Met306Ile	pncA_p.Ala146Val	gyrB_p.Ala504Val	NI	NI	NI
EUST20722	XDR	rpoB_p.Ser450Leu	inhA_c.-777C > T, katG_p.Ser315Thr	embB_p.Met306Ile	pncA_p.Ala146Val	gyrA_p.Asp94Tyr	Rv0678_lof(778990:Del-long)	NI	NI
EUST28527	Pre-XDR	rpoB_p.Ser450Leu	inhA_c.-777C > T, katG_p.Ser315Thr	embB_p.Met306Ile	pncA_p.Ala146Val	gyrA_p.Asp94Tyr	Rv0678_p.Gly25Cys^a^	NI	NI
EUST19926	XDR	rpoB_p.Ser450Leu	inhA_c.-777C > T, katG_p.Ser315Thr	embB_p.Met306Ile	pncA_p.Ala146Val	gyrA_p.Asp94Tyr	Rv0678_lof(778990:Del-long)	NI	NI
IT3467	Pre-XDR	rpoB_p.Ser450Leu	inhA_c.-777C > T, katG_p.Ser315Thr	embB_p.Met306Ile	pncA_p.Ala146Val	gyrA_p.Asp94Tyr	NI	NI	NI
EUST26488	XDR	rpoB_p.Ser450Leu	inhA_c.-777C > T, katG_p.Ser315Thr	embB_p.Met306Ile	pncA_p.Ala146Val	gyrA_p.Asp94Tyr	Rv0678_lof(778990:Del-long)	NI	NI
EUST07876	XDR	rpoB_p.Ser450Leu	inhA_c.-777C > T, katG_p.Ser315Thr	embB_p.Met306Ile	pncA_p.Ala146Val	gyrA_p.Asp94Tyr	Rv0678_lof(778990:Del-long)	NI	NI
IT4121	XDR	rpoB_p.Ser450Leu	inhA_c.-777C > T, katG_p.Ser315Thr	embB_p.Met306Ile	pncA_p.Ala146Val	gyrA_p.Asp94Tyr	Rv0678_lof(778990:Del-long)	NI	NI
IT4315	XDR	rpoB_p.Ser450Leu	inhA_c.-777C > T, katG_p.Ser315Thr	embB_p.Met306Ile	pncA_p.Ala146Val	gyrA_p.Asp94Tyr	Rv0678_lof(778990:Del-long)	NI	NI
IT4244	XDR	rpoB_p.Ser450Leu	inhA_c.-777C > T, katG_p.Ser315Thr	embB_p.Met306Ile	pncA_p.Ala146Val	gyrA_p.Asp94Tyr	Rv0678_lof(778990:Del-long)	NI	NI
IT4734	Pre-XDR	rpoB_p.Ser450Leu	inhA_c.-777C > T, katG_p.Ser315Thr	embB_p.Met306Ile	pncA_p.Ala146Val	gyrA_p.Asp94Tyr	Rv0678_Arg34Leu^b^	NI	NI
IT4735	XDR	rpoB_p.Ser450Leu	inhA_c.-777C > T, katG_p.Ser315Thr	embB_p.Met306Ile	pncA_p.Ala146Val	gyrA_p.Asp94Tyr	Rv0678_lof(779005:Del-1); Rv0678_lof(779182:Del-1)	NI	NI
IT4737	XDR	rpoB_p.Ser450Leu	inhA_c.-777C > T, katG_p.Ser315Thr	embB_p.Met306Ile	pncA_p.Ala146Val	gyrA_p.Asp94Tyr	Rv0678_lof(778990:Del-long)	fbiC_stop_668Gly	NI

MDR: multidrug-resistant tuberculosis; NI: no WHO mutation catalogue, version 2 mutations of group 1, 2, 3 were identified, or mutations of possible interest in the WHO mutation catalogue Tier 1 genes; WHO: World Health Organization; XDR: extensively drug-resistant tuberculosis.

^a^ Mutations belong to group 3 (mutations of uncertain significance).

^b^ Determinants were not present in version 2 of the WHO mutation catalogue, version 2 [10], nor in any available systematic review.

^c^ Mutation was acquired in 2024, 6 years after the first isolation.

The samples were analysed using a pipeline developed in-house that integrates MTBseq (v1.0.4) [8] and polishes analysis for structural variations, such as insertions and deletions, with Delly (v1.1.6) [9]. The reported resistance-associated mutations belong to groups 1 and 2 according to the WHO mutation catalogue, version 2 [10]. Samples are presented from top to bottom in order of identification.

Twenty-five isolates carried mutations in the *gyrA* and *gyrB* genes. The earliest mutation detected within the cluster was *gyrB* p.Ala504Val, first identified in 2017 in Romania and, in the same year, in Italy in an individual of Albanian origin. The same mutation was subsequently reported in a locally born case in Romania, and in another case detected in Italy in a person born in Romania. A single isolate identified in 2017 from a Romanian-born individual carried *gyrA* p.Asp94Gly. Notably, this was the only case presenting resistance to amikacin (rrsA1401G mutation). All remaining pre-XDR and XDR cases (n = 18) carried *gyrA* p.Asp94Tyr, the main fluoroquinolone resistance determinant in the cluster.

Among the 15 XDR isolates, all identified in Italy between 2018 and 2025, resistance to bedaquiline was linked to loss-of-function mutations in Rv0678, including a large deletion at position 778,990 (n = 13) and smaller deletions at positions 779,005 (n = 1) and 779,182 (n = 1). Moreover, two isolates carried the p.Gly25Cys mutation in Rv0678 (one alone and one with a 778,990 deletion), classified as ‘of uncertain significance’ according to the WHO catalogue [10]. Moreover, two isolates presented a mutation not reported in the WHO catalogue (Rv0678_p.Arg34Leu) nor in the available systematic reviews on Rv0678 mutations associated with bedaquiline resistance [11,12]. The available phenotypic data confirm resistance to bedaquiline for the isolate with the solo Rv0678 p.Gly25Cys mutation, as well as for the most recent case isolated in July 2025 carrying the p.Arg34Leu mutation on Rv0678. In both cases, minimum inhibitory concentrations (MICs) were determined using BD BACTEC mycobacteria growth indicator tube (MGIT) detection system (Becton, Dickinson, and company) with a result of 4 mg/L.

Furthermore, strain IT4737, isolated in August 2025, showed resistance to pretomanid and delamanid linked to a loss-of-function mutation in *fbiC*. The individual had no previous TB treatment, and contact tracing did not reveal any link to known cases. Notably, a mutation in the same gene (*fbiC*_p.His722Arg) emerged in 2024 in the latest isolate of one of the established cluster cases. Only 2 SNPs separate the two isolates, indicating a close genomic relationship. Even though the mutation p.His722Arg is not listed in the WHO Catalogue, drug-susceptibility testing in MGIT confirmed resistance to both delamanid and pretomanid.

## Discussion

The ongoing transmission and increasing resistance development of MTB cluster snpCL1 reflects a progression from MDR to XDR since its initial detection within the EUSeqMyTB project and driven by sequential acquisition of resistance-conferring mutations in *gyrA*, *gyrB*, Rv0678 and *fbiC*. The limited availability of sequencing data in the TESSy database hampers the assessment of whether transmission continues in other countries, highlighting gaps in genomic surveillance that can limit understanding of transmission and the emergence of resistant variants [13]. 

Whole genome sequencing plays a crucial role in identifying cross-border clusters tracking transmission, and monitoring the spread and development of resistance [2,14]. The EUSeqMyTB project demonstrated that WGS strengthens epidemiological surveillance by determining the genetic relatedness of isolates, facilitating contact tracing, and supporting coordinated public health action across borders [3,15]. Further studies confirm that systematic WGS implementation offers both clinical and public health benefits in low TB incidence settings where WGS shortens turnaround time compared with phenotypic drug susceptibility testing, while providing comprehensive resistance profiles, enabling outbreak investigations, and potentially reducing management costs [16-19].

## Conclusion

*Mycobacterium tuberculosis* clone snpCL1’s rapid adaptive evolution from MDR to XDR poses a notable risk to TB elimination goals in Europe, particularly if it becomes more transmissible. Continuous, coordinated WGS surveillance across EU/EEA countries, supported by timely data sharing and integration with epidemiological investigations, is essential to identify ongoing transmission early and prevent the further spread of highly resistant MTB clones in Europe.

## Data Availability

These data are not publicly available in compliance with ECDC guidelines. WGS data should be requested through this portal: https://www.ecdc.europa.eu/en/publications-data/access-eueea-surveillance-data-third-parties

## Disclaimer

The views and opinions of the authors expressed herein do not necessarily state or reflect those of ECDC. The accuracy of the authors’ analysis and the findings they report are not the responsibility of ECDC. ECDC is not responsible for conclusions or opinions drawn from the data provided. ECDC is not responsible for the correctness of the data and for data management, data merging and data collation after provision of the data. ECDC shall not be held liable for improper or incorrect use of the data.

## Supplementary Data

342000 Supplement
