# Supplementary material for: Tracking the evolution of an extensively drug-resistant cross-border Mycobacterium tuberculosis cluster, Europe, January 2016 up to August 2025: implications for European surveillance
Source: Euro Surveill. 2025 Nov 20;30(46):2500838. doi: 10.2807/1560-7917.ES.2025.30.46.2500838 (PMC12639277; doi:10.2807/1560-7917.ES.2025.30.46.2500838)

Tracking the evolution of an extensively resistant cross-border tuberculosis cluster, Europe, January 2016 up to August 2025: implications for European surveillance

Supplementary Materials

This supplementary material is hosted by Eurosurveillance as supporting information alongside the article *Tracking the evolution of an extensively resistant cross-border tuberculosis cluster, Europe, January 2016 up to August 2025: implications for European surveillance* on behalf of the authors who remain responsible for the accuracy and appropriateness of the content. The same standards for ethics, copyright, attributions and permissions as for the article apply. Eurosurveillance is not responsible for the maintenance of any links or email addresses provided therein.

Supplementary Table S1. Cross-border clusters identified using SeqSphere (cgMLST), with a ≤ 5-allele difference threshold, with the number of samples per cluster and countries involved

Acronyms: AT,Austria; BE,Belgium; BG,Bulgaria; CZ,Czech Republic; DE,Germany; DK,Denmark; EE,Estonia; ES,Spain; FI,Finland; FR,France; HR,Croatia; HU,Hungary; IE,Ireland; IT,Italy; LT,Lithuania; LV,Latvia; NL,Netherlands; NO,Norway; PL,Poland; PT,Portugal; RO,Romania; SE,Sweden; SI,Slovenia; SK,Slovakia; UK,United Kingdom

Cluster ID	Number of samples per cluster	Countries involved (number of samples per country)
1	145	LT (83), EE (47), DE (6), CZ (2), FI (1), FR (1), HU (1), IE (1), LV (1), PL (1), UK (1)
2	95	RO (61), IT (30), UK (2), AT (2)
3	36	LT (34), NO (1), UK (1)
4	32	LT (28), IE (2), PL (2)
5	32	LT (31), UK (1)
6	30	PL (15), IT (5), CZ (2), DE (1), EE (1), FR (2), BE (1), NL (1), LT (1), SK (1)
7	20	RO (19), DE (1)
9	19	DE (7), IT (7), AT (4), FR (1)
10	14	RO (13), DE (1)
11	13	RO (11), IT (2)
12	12	CZ (3), EE (3), DE (1), ES (1), FI (1), PL (2), SE (1)
14	12	RO (11), DE (1)
15	12	DE (5), IT (5), NL (1), SE (1)
16	11	RO (9), BE (1), DE (1)
17	11	IT (8), ES (3)
18	10	ES (9), SE (1)
19	10	RO (8), IT (2)
20	10	HU (9), IT (1)
21	9	EE (8), FI (1)
22	9	IT (3), SE (3), DE (1), FR (1), UK (1)
23	9	LV (4), EE (3), FR (1), NL (1)
25	8	LV (4), EE (2), IE (1), LT (1)
27	8	DE (4), ES (1), IT (1), LV (1), PL (1)
33	7	PL (6), EE (1)
36	6	BG (5), SI (1)

39	6	LT (5), UK (1)
40	6	LT (5), UK (1)
42	6	DE (3), ES (1), FR (1), IT (1), AT (1)
43	6	ES (3), LT (3)
45	6	IT (4), ES (1), NL (1)
52	5	IT (4), RO (1)
57	5	AT (3), DE (2)
59	5	RO (4), FR (1)
61	5	LT (4), IE (1)
77	4	BG (2), DE (2)

Supplementary Table S2. National clusters identified using SeqSphere (cgMLST) with a ≤ 5-allele difference threshold, with the number of samples per cluster and countries involved

Acronyms: BG,Bulgaria; DE,Germany; DK,Denmark; EE,Estonia; ES,Spain; FR,France; HU,Hungary; LT,Lithuania; LV,Latvia; PL,Poland; PT,Portugal; RO,Romania

Cluster ID	Number of samples per cluster	Countries involved (number of samples per country)
8	20	RO (20)
13	12	RO (12)
24	9	LT (9)
26	8	LT (8)
28	8	LT (8)
29	8	RO (8)
30	8	PT (8)
31	7	RO (7)
32	7	RO (7)
34	6	HU (6)
35	6	LT (6)
37	6	BG (6)
38	6	LV (6)
41	6	BG (6)
44	6	RO (6)
46	6	RO (6)
47	6	EE (6)
48	6	RO (6)
49	5	LT (5)
50	5	RO (5)
51	5	LT (5)
53	5	LV (5)
54	5	LT (5)
55	5	RO (5)
56	5	RO (5)
58	5	PL (5)
60	5	RO (5)
62	5	BG (5)
63	5	DK (5)
64	5	LT (5)
65	5	RO (5)

66	5	RO (5)
67	5	RO (5)
68	4	FR (4)
69	4	ES (4)
70	4	BG (4)
71	4	BG (4)
72	4	RO (4)
73	4	DE (4)
74	4	LT (4)
75	4	RO (4)
76	4	RO (4)
78	4	RO (4)
79	4	LT (4)

Supplementary Figure S1. Cross-border cluster comprising 94 *Mycobacterium tuberculosis* complex strains identified using SeqSphere (cgMLST) with a ≤ 5-allele difference threshold

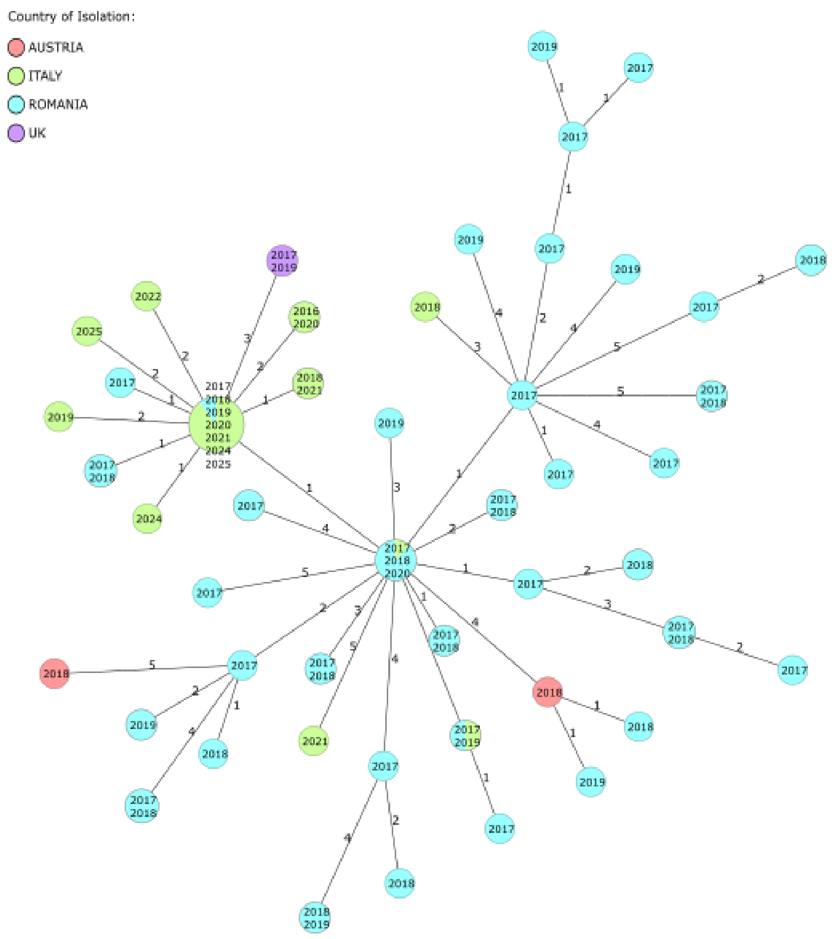

Supplement: Supplement [file 25-00838_CIRILLO_Supplement.pdf]
